# Assessment of environmental and carcinogenic health hazards from heavy metal contamination in sediments of wetlands

**DOI:** 10.1038/s41598-023-43349-7

**Published:** 2023-09-28

**Authors:** Bibhu Prasad Panda, Yugal Kishore Mohanta, Rakesh Paul, B. Anjan Kumar Prusty, Siba Prasad Parida, Abanti Pradhan, Muthupandian Saravanan, Kaustuvmani Patowary, Guangming Jiang, Sanket J. Joshi, Hemen Sarma

**Affiliations:** 1 Salim Ali Centre for Ornithology and Natural History, South India Centre of Wildlife Institute of India, Coimbatore, Tamil Nadu 641108 India; 2 Environmental Sciences, Department of Chemistry and BBRC, ITER, Siksha ‘O’ Anusandhan (Deemed to be University), Bhubaneswar, Odisha 751030 India; 3 Nano-biotechnology and Translational Knowledge Laboratory, Department of Applied Biology, School of Biological Sciences, University of Science and Technology Meghalaya, Ri-Bhoi, Meghalaya 793101 India; 4 Centre for Herbal Pharmacology and Environmental Sustainability, Chettinad Hospital, and Research Institute, Chettinad Academy of Research and Education, Kelambakkam, Tamil Nadu 603103 India; 5 Department of Biodiversity and Conservation of Natural Resources, Central University of Odisha, Koraput, Odisha 764021 India; 6 Department of Environmental Studies, Berhampur University, Berhampur, Odisha 760007 India; 7 Department of Zoology, School of Applied Sciences, Centurion University of Technology and Management, Bhubaneswar, Odisha 752050 India; 8 AMR and Nanomedicine Laboratory, Department of Pharmacology, Saveetha Dental College, Saveetha Institute of Medical and Technical Sciences (SIMATS), Chennai, 600077 India; 9 School of Civil, Mining, Environmental and Architectural Engineering, University of Wollongong, Wollongong, NSW 2522 Australia; 10 Oil & Gas Research Centre, Central Analytical and Applied Research Unit, Sultan Qaboos University, Muscat, Oman; 11 Bioremediation Technology Group, Department of Botany, Bodoland University, Rangalikhata, Deborgaon, Kokrajhar (BTR), Assam 783370 India

**Keywords:** Cancer, Ecology, Biogeochemistry, Environmental sciences, Natural hazards, Risk factors

## Abstract

Sediment contamination jeopardizes wetlands by harming aquatic organisms, disrupting food webs, and reducing biodiversity. Carcinogenic substances like heavy metals bioaccumulate in sediments and expose consumers to a greater risk of cancer. This study reports Pb, Cr, Cu, and Zn levels in sediments from eight wetlands in India. The Pb (51.25 ± 4.46 µg/g) and Cr (266 ± 6.95 µg/g) concentrations were highest in Hirakud, Cu (34.27 ± 2.2 µg/g) in Bhadrak, and Zn (55.45 ± 2.93 µg/g) in Koraput. The mean Pb, Cr, and Cu values in sediments exceeded the toxicity reference value. The contamination factor for Cr was the highest of the four metals studied at Hirakud (CF = 7.60) and Talcher (CF = 6.97). Furthermore, high and moderate positive correlations were observed between Cu and Zn (r = 0.77) and Pb and Cr (r = 0.36), respectively, across all sites. Cancer patients were found to be more concentrated in areas with higher concentrations of Pb and Cr, which are more carcinogenic. The link between heavy metals in wetland sediments and human cancer could be used to make policies that limit people's exposure to heavy metals and protect their health.

## Introduction

Wetlands have had a long and crucial connection to human civilization since ancient times, rendering multiple benefits and services to humans^1^. The wetland ecosystem supports the hydrological cycle, regulates climate change, and provides many ecosystem services to biodiversity^2^. In addition, it adds direct and indirect value to human beings by supporting various economic services^3^. Considering the land area as a unit, the wetland ecosystem can be described as a top ecosystem service that provides 47% of the global ecosystem value^4^. This fact makes this ecosystem vital and fruitful among all ecosystems^2^. This ecosystem is also a heavy metal sink due to its importance and role in several physical, chemical, and biological events. In the modern world, anthropogenic activity serves the most to deposit heavy metals in this sink^5–7^. The typical heavy metal pollutants produced through urbanization, industrialization, and agricultural practices are lead (Pb), chromium (Cr), cadmium (Cd), copper (Cu), mercury (Hg), nickel (Ni), zinc (Zn), manganese (Mn), and arsenic (As)^8^.

Heavy metals could be present in soils in various concentrations, indicating either natural lithogenic sources or anthropogenic processes^9^. Heavy metal concentrations that are too high^10^ and other necessary and non-essential components in aquatic habitats^11^ can indicate the inputs from the catchment and surrounding area, and different indices can be employed to measure the contamination level^12^. Cd, Cr, and Pb are all hazardous to all creatures. Metals like Cu, Zn, and Mn are thought necessary for their function in biochemical functioning in organisms, but they are also known to be harmful beyond the threshold limit^13–15^. The higher tendencies for bioaccumulation make them biologically harmful^16,17^. These metals are continuously deposited in water and sediment in any given habitat, eventually leading to accumulation in different organisms inhabiting the particular habitat^18,19^; determining metal concentrations in the habitat is essential^10^ in evaluating the contamination profiles.

Heavy metal concentrations in bottom sediment have been used to indicate environmental pollution in different ecosystems, viz., rivers^20,21^, streams^22^, wetlands^23–26^, forests^27^, grasslands^28^, and marine ecosystems^29^. The heavy metal load in bottom sediments in wetlands can indicate both natural sources and human-caused activities, as industrial waste channelled through streams, rivers, and agricultural runoff^23,30–32^. Because of their tenacity and increased intensity in agriculture^7,33,34^, heavy metals accumulate in wetland soil over the years, posing threats to the environment and human well-being as they flow through the trophic levels^35,36^.

The flow of heavy metals from soil to livestock and humans can occur either by directly consuming tainted crops or bioaccumulation through the food chain^37,38^. Such processes are driven by several factors known to have distinct spatiotemporal variability. Thus, the existing understanding of metal distribution, sources, and contamination risk in wetlands must be supplemented with additional findings from different types of wetlands spread across varied landscapes. The environmental quality of wetlands can be judged using sedimentary heavy metal content as an indicator^39–41^. Analyzing and assessing heavy metal concentrations has become essential to monitoring wetland pollution^42,43^. Knowledge of the intensity of contamination can be gained by assessing different sediment qualities^44–46^. Only a few studies have investigated the content of heavy metals in the soil in this study area^47–50^. In India, few studies can indicate wetland health from metal contamination and the accompanying human health risk.

Because of these specifics, the current investigation was conducted to: (i) examine the accumulation of Pb, Cr, Cu, and Zn in the soil of wetlands with distinct spatial distribution in Odisha, India; (ii) make an ecological risk assessment of wetlands inside agricultural landscapes; and (iii) evaluate the human health risk potential of Pb and Cr. The expected outcome of this study is to show heavy metal pollution's influence on wetland health and the risk to human health.

## Methods

### Study area and sampling site

**Figure 1:**
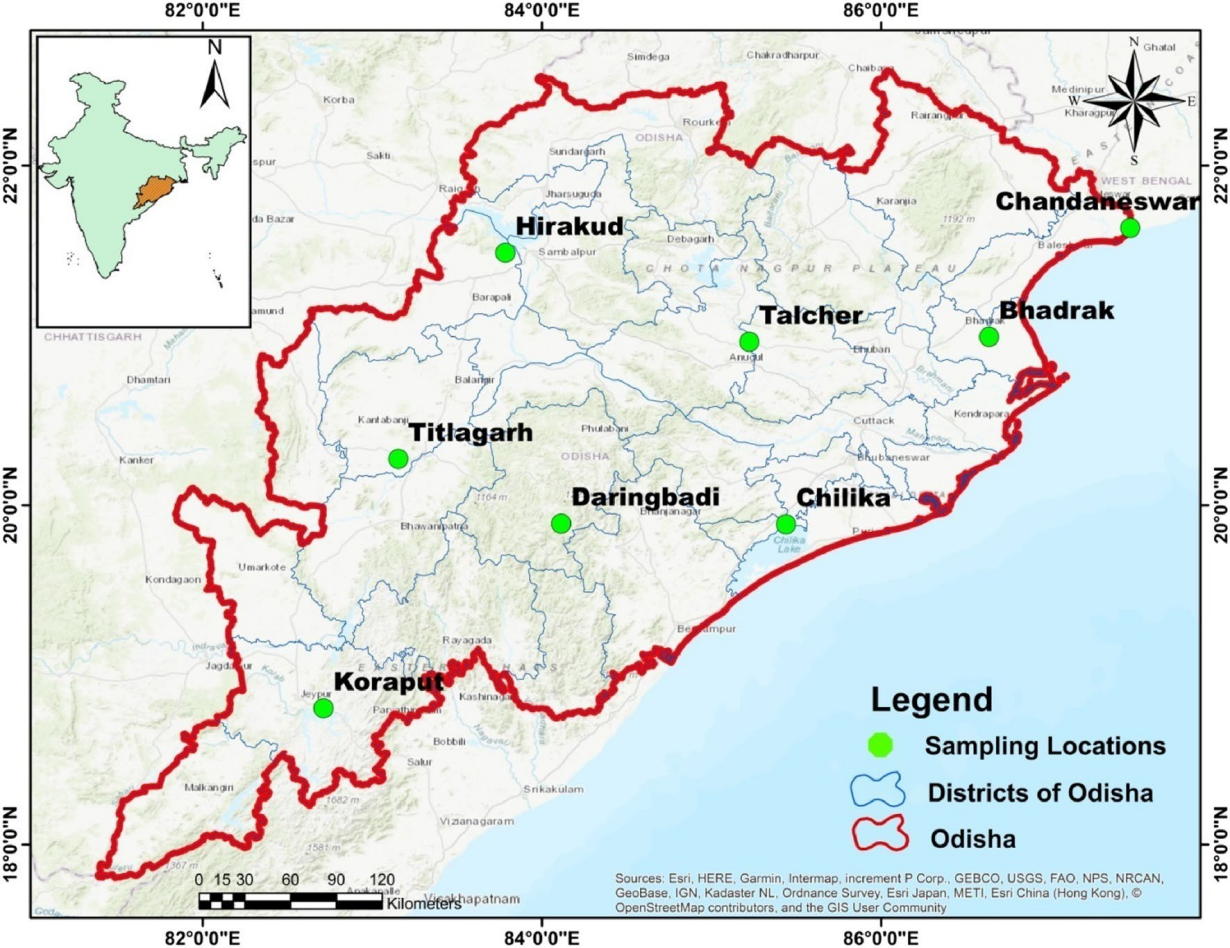
Map of the study region with sampling sites crated using ArcMap 10.2.1.

The present study covered eight different wetlands in the Indian state of Odisha, located in distinct landscapes and with distinct sources of contamination (Fig. 1). Of the eight wetlands, Chandaneswar, Chilika, Daringbadi, and Koraput are natural wetlands, and Bhadrak, Hirakud, Talcher, and Titlagarh are constructed wetlands. The details of the location characteristics of the wetlands are presented in Table 1.

**Table 1:** Details of location characteristics of sampling points of the study area.

Wetland	Type	Location	Land use pattern	Sources of pollutants
Bhadrak	Constructed	Bhadrak	Agricultural wetland with seasonal agriculture	Insecticide, pesticide from agricultural runoff
Chandaneswar	Natural	Balasore	Agricultural land covered by year-wide crops	Agricultural runoff, anthropogenic activity
Chilika	Natural	Khurda	The anthropogenic activity primarily by cattle	Agricultural runoff, village runoff, anthropogenic activity
Daringbadi	Natural	Kandhamal	Undisturbed natural water body	Natural sources
Hirakud	Constructed	Bargarh	Agricultural activity covering the whole year	Natural sources, agricultural runoff
Koraput	Natural	Koraput	Connected to a reservoir in monsoon and separated in other seasons, anthropogenic activity	Natural sources, cattle grazing
Talcher	Constructed	Angul	Urban area, anthropogenic activity	Urban pollution, industrial pollution, coal mining
Titlagarh	Constructed	Bolangir	Semi-urban area, anthropogenic activity	Urban pollution, anthropogenic activity

### Sediment sampling

Bed sediment samples (in triplicate) were collected every other month between October 2015 and August 2018 using the grab sampling technique^26^. In total, 144 samples were collected from the eight identified wetlands. Bed sediment samples, collected from 5 to 10 cm depth, air-dried in the laboratory after being transported in resealable polythene bags, followed by oven-drying at 50–60 °C until constant weight, and homogenized using a mortar and pestle^51^. Finally, the homogenized samples were sieved using a 2 mm mesh sieve before being placed in clean plastic containers^52^.

### Sample digestion

One gram of powdered sediment sample was transferred to a Teflon digester tube in a microwave digestion system (Milestone, MLS 1200), which was programmed to have the sequential addition of a series of acids, i.e. 10 ml HNO_3_ for 10 min, 1 ml HClO_4_ for 5 min, and 5 ml H_2_O_2_ for 10 min, at 250W magnetron power settings^29^. A digestion blank without a sample was also included. By adding deionized water, the digested samples were filtered, made up to 50 ml, and stored in pre-cleaned and acid-treated plastic vials at 4°C^53^.

### Sample analysis

The concentration of heavy metals in the digested samples was detected utilizing a double-beam atomic absorption spectrophotometer (Shimadzu, AA 6300) under standard analytical conditions. The detection limits (DL) for Pb, Cr, Cu, and Zn were 0.03 µg/g, 0.02 µg/g, 0.002 µg/g, and 0.02 µg/g, respectively. The standard addition technique was used to reduce the matrix effects in the analyses. As part of the QA/QC process, pre-analyzed soil samples were used as reference material subjected to the same analytical methods for estimating the detection limits of the metals^54^.

### Contamination indices of pollution

Both the Contamination Factor (CF) and Geo-accumulation Index (Igeo) are widely used to assess the contamination level in wetlands, and they provide essential information for comprehending the effects of pollution on these ecosystems. The CF is a measure used to assess the level of contamination in a specific environment, such as wetlands^55^. It is calculated by comparing the concentration of an element in the sediment to its background value in the environment.

1 $${\text{CF }} = {\text{ C}}_{{\text{S}}} /{\text{C}}_{{\text{B}}} .$$

C_S_ = element concentration (µg/g) in the analyzed sediment, and CB = element (µg/g) in the reference background. The background values of the elements used are Pb (20), Cr (35), Cu (25), and Zn (71)^8^.

The Geo-accumulation index (Igeo) is another used to assess wetland contamination. It measures the accumulation of a specific element in the sediment relative to its background concentration in the environment. The formula can be used to compute it as proposed below^56^.

2 $${\text{I}}_{{{\text{geo}}}} = {\text{ log}}_{{2}} \left( {{\text{C}}_{{\text{S}}} /{1}.{\text{5C}}_{{\text{B}}} } \right).$$

The descriptions for C_S_ and C_B_ have been provided earlier. The I_geo_ comprises 7 grades in the 5 < Igeo ≤ 0 57–59 range. The grades are I_geo_ ≤ 0 (soil is not contaminated); 0 < I_geo_ ≤ 1 (uncontaminated up to moderately contaminated); 1 < I_geo_ ≤ 2 (moderately contaminated); 2 < I_geo_ ≤ 3 (moderately up to strongly contaminated); 3 < I_geo_ ≤ 4 (strongly contaminated); 4 < I_geo_ ≤ 5 (strongly up to extremely contaminated); and lastly I_geo_ > 5 (extremely contaminated)^57^.

### Ecological risk assessment

Two indices, the potential ecological risk factor (PERF) and the potential ecological risk index (PERI or RI), were used to conduct the ecological risk assessment. The PERF can describe the contamination due to one element (heavy metal). It can be calculated using the formula.

3 $${\text{PERF }} = {\text{ CF }} \times {\text{ TRF}}.$$

CF represents the contamination factor for each element/heavy metal, and TRF represents the toxicological response factor. The TRF for the detected elements/heavy metals is Pb:5, Cr:2, Cu:5, and Zn:1^8,45,58^. This formula resonates with the hazards to humans and the ecosystem and the ecological vulnerability to heavy metal contamination^59^. Further, the PERI or RI describes the total potential risk presented by all the components found in the sediment^55^, which was empirically estimated by summing up all the PREF values obtained for each element using the following equation proposed by^58^:

4 $${\text{RI }} = \, \sum {\text{PERF}}.$$

RI represents the potential ecological risk index of all detected elements, and PERF represents the individual elements’ potential ecological risk index.

### Human health risk assessment

The relationship between the ecosystem, human health, and contaminants in the environment can be assessed by assessing the human health risk using the guidelines of USEPA^60^. The present study assesses carcinogenic and non-carcinogenic risks via ingestion pathways. Health risk levels may be site-specific due to exposure to an element (heavy metals). The average daily dose (ADD) can be calculated to identify non-carcinogenic threats. The ADD by ingestion was calculated as follows:

5 $${\text{ADD }} = \, \left( {{\text{Cs }} \times {\text{ IR }} \times {\text{ EF }} \times {\text{ ED}}} \right)/\left( {{\text{BW}}/{\text{AT}}} \right),$$

where C_S_ is the concentration of heavy metal (µg/g) in analyzed sediment; IR is the ingestion rate of contaminated sediment (0.001 kg/day for children and 0.0035 kg/day for an adult); EF is the exposure frequency (300 days/year, assumed); ED is the exposure duration (6 years for children and 30 years for an adult); BW is the body weight (15 kg for children and 70 kg for an adult), and AT is the average time (2190 days for children and 10,950 days for an adult^61^.

Using the hazard quotient (HQ), the non-carcinogenic harmful effects of heavy metals were measured^62^. The HQ value was estimated as follows:

6 $${\text{HQ }} = {\text{ ADD }}/{\text{ RfD}}.$$

The average daily dose is ADD; RfD is the equivalent reference dose. The RfD values for the detected metals/elements are Pb:0.0035 µg/g; Cr:1.5 µg/g; Cu:0.04 µg/g and Zn:0.3 µg/g^8^. The hazard index (HI) can determine the full carcinogenic effect, which can be calculated by adding all ‘metals’ HQ to this formula^34^.

7 $${\text{HI }} = {\text{ HQ}}_{{1}} + {\text{ HQ}}_{{2}} + {\text{ HQ}}_{{3}} + \, \ldots + {\text{ HQ}}_{{\text{n}}} ,$$

In addition to the non-carcinogenic effects, humans exposed to contaminated sediment can face carcinogenic risk (CR) their whole lives. The CR can be measured by this formula^58^:

8 $${\text{CR }} = {\text{ ADD }} \times {\text{ SF}}.$$

ADD is the average daily dose, and SF is the slope factor of the respected element/heavy metal. The SF used in this study for Pb is 0.042, and for Cr is 0.5, according to the US Environmental Protection Agency. However, the other two metals are not listed due to their less carcinogenic effects^8^.

### Spatial distribution of data

In a given geographical framework, interpolating spatial parameters utilizing tools like the Geographic Information System (GIS) integrating field inventory has provided agility in scientific representation^63^. IDW interpolation method was used in ArcMap 10.2.1's Spatial Analyst Tools to depict the contamination's spatial distribution. No minimum number of points was set, and the output cell size was taken as 0.01 to get a smooth prediction of the values in the unsampled/unmeasured areas and give a detailed account of how each parameter is distributed spatially compared to the others. The neighborhood was taken as 12, the optimal number for eight sampling locations. However, the maximum distance for the search radius was kept as the default because all the parameters are static, and there are no directional influences.

### Statistical analysis

The datasets were subjected to an appropriate suite of statistical tests. Descriptive statistics determined the range, median, and average values. First, a two-way Pearson correlation test was conducted to determine the connection between the various metals in the soil. The significant difference in heavy metals and wetlands concerning sediment was tested using a one-way analysis of Variance (ANOVA). Second, hierarchical cluster analysis was conducted to identify the system of organized variables where the same clusters share common data properties. The significance level for the statistical tests was α = 0.05 for all analyses.

## Results and discussion

### Heavy metal concentration in sediment

**Figure 2:**
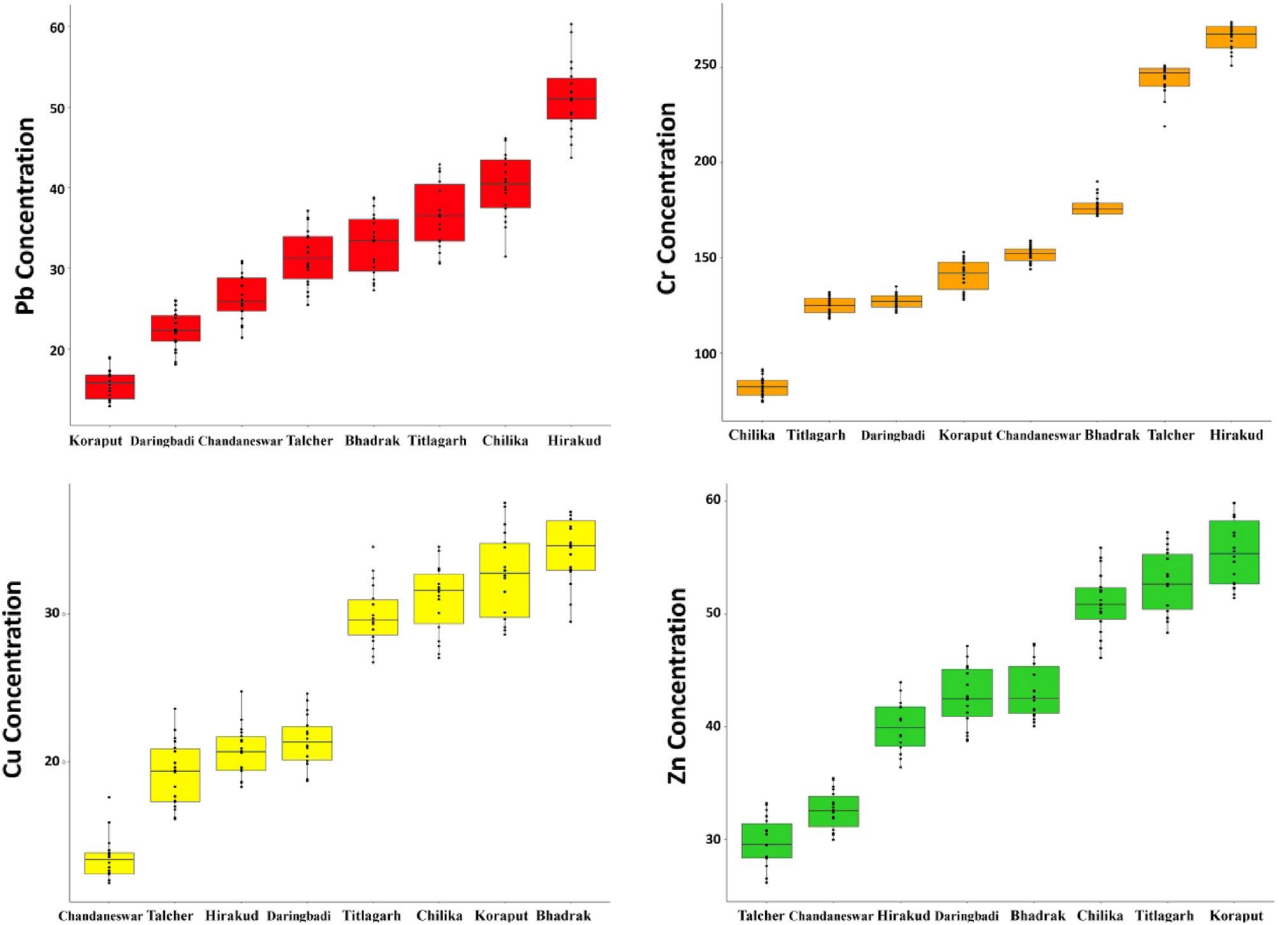
Concentrations of heavy metals (µg/g) in each of the locations in ascending order.

The concentrations of Pb, Cr, Cu, and Zn recorded in bed sediments are presented in Table 2. The Pb concentration was the highest at the Hirakud sampling site (51.25 ± 4.46 µg/g), and all sampling sites recorded higher concentrations of Pb than previous studies^47,49^. The concentration of Pb was found to be significantly different among sites (F = 177.4, P < 0.001). The Cr was the highest at the Hirakud sampling site (266 ± 6.95 µg/g), much higher than previous studies from Odisha^48–50^. The concentration of Cr was found to be significantly different among sites (F = 1911, P < 0.001). The highest Cu concentration was recorded at the Bhadrak site (34.27 ± 2.2 µg/g), and all other sampling sites, except Chandaneswar, also recorded higher concentrations of Cu than previous studies^47–49^. The concentration of Cu was found to be significantly different among sites (F = 226.4, P < 0.001). The mean concentration of Zn was discovered to be the most abundant at Koraput (55.45 ± 2.93 µg/g), which is unlikely to be lower than previous studies in Odisha^47–49^. The concentration of Zn was determined to be distinguishable in a significant manner (F = 245.1, P < 0.001) among all sites (Fig. 2). Comparisons have been made between the concentrations of heavy metals measured at each sampling location and the international standards and threshold levels specified by different agencies (Table 2). A list of metal and sampling locations in decreasing order is presented in Table 3. All the sites recorded higher Cr concentrations than other detected metals. The natural wetlands had Cr, Zn, and Pb in decreasing order, while the constructed wetlands had higher Cr followed by Pb and Zn, respectively (Table 3).

**Table 2:** Descriptive statistics of recorded heavy metal concentrations in different locations (N = 144).

Sampling sites	Pb	Cr	Cu	Zn
Bhadrak	27.26–38.74^a^	172–190	29.46–36.9	40.05–47.37
	32.88 ± 3.85^b^	177 ± 5.19	34.27 ± 2.2	43.18 ± 2.55
	0.91^c^	1.22	0.52	0.6
	14.82^d^	26.94	4.83	6.48
Chandaneswar	21.38–30.91	144–159	11.82–17.6	29.94–35.42
	26.44 ± 2.93	152 ± 4.24	13.43 ± 1.49	32.52 ± 1.76
	0.69	1	0.35	0.42
	8.6	18	2.22	3.11
Chilika	31.46–46.13	74.26–91.3	27.02–34.51	46.11–55.91
	40.03 ± 4.14	82.05 ± 5.35	31 ± 2.3	50.99 ± 2.73
	0.98	1.26	0.54	0.64
	17.17	28.6	5.31	7.44
Daringbadi	18.03–25.98	121–135	18.69–24.59	38.75–47.18
	22.32 ± 2.43	127 ± 3.93	21.38 ± 1.78	42.69 ± 2.7
	0.57	0.93	0.42	0.64
	5.93	15.41	3.18	7.3
Hirakud	43.72–60.3	251–274	18.27–24.74	36.39–43.94
	51.25 ± 4.46	266 ± 6.95	20.63 ± 1.74	40.08 ± 2.34
	1.05	1.64	0.41	0.55
	19.87	48.35	3.03	5.48
Koraput	12.78–18.94	128–153	28.58–37.5	51.42–59.9
	15.55 ± 1.9	141 ± 8.18	32.55 ± 2.96	55.45 ± 2.93
	0.45	1.93	0.7	0.69
	3.62	66.94	8.77	8.59
Talcher	25.43–37.1	219–251	16.15–23.57	26.14–33.21
	31.35 ± 3.51	244 ± 8.22	19.18 ± 2.22	29.71 ± 2.26
	0.83	1.94	0.52	0.53
	12.29	67.53	4.91	5.11
Titlagarh	30.27–42.9	118–132	26.72–34.53	48.33–57.29
	36.63 ± 4.14	125 ± 4.65	29.93 ± 2.08	52.87 ± 2.77
	0.98	1.1	0.49	0.65
	17.14	21.65	4.31	7.67
BV^e^	20	35	25	71
TVAS^f^	60	110	63	200
TRV^g^	31	26	16	110
WCTMRL^h^	10–100	20–190	20–90	50–250

^a^Concentration ranges (µg/g), ^b^Mean ± Standard deviation (SD) (µg/g), ^c^Standard error (SE), ^d^Variance, ^e^Background value (BV)^12^, ^f^Threshold values for agricultural soil (TVAS)^64^, ^g^Toxicity reference value (TRV)^65^, ^h^World Common Trace Metal Range in Lake (WCTMRL) sediment^66^.

**Table 3:** List of metals and sampling locations in decreasing order.

Type	Location	Metal concentration
Constructed	BDRK	Cr > Zn > Cu > Pb
Natural	CDSR	Cr > Zn > Pb > Cu
Natural	CHLK	Cr > Zn > Pb > Cu
Natural	DRBD	Cr > Zn > Pb > Cu
Constructed	HRKD	Cr > Pb > Zn > Cu
Natural	KRPT	Cr > Zn > Cu > Pb
Constructed	TLHR	Cr > Pb > Zn > Cu
Constructed	TTGH	Cr > Zn > Pb > Cu

Metal	Locations	
Pb	HRKD > CHLK > TTGH > BDRK > TLHR > CDSR > DRBD > KRPT	
Cr	HRKD > TLHR > BDRK > CDSR > KRPT > DRBD > TTGH > CHLK	
Cu	BDRK > KRPT > CHLK > TTGH > DRBD > HRKD > TLHR > CDSR	
Zn	KRPT > TTGH > CHLK > BDRK > DRBD > HRKD > CDSR > TLHR	

*BDRK* Bhadrak, *CDSR* Chandaneswar, *CHLK* Chilika, *DRBD* Daringbadi, *HRKD* Hirakud, *KRPT* Koraput, *TLHR* Talcher, *TTGH* Titlagarh.

**Figure 3:**
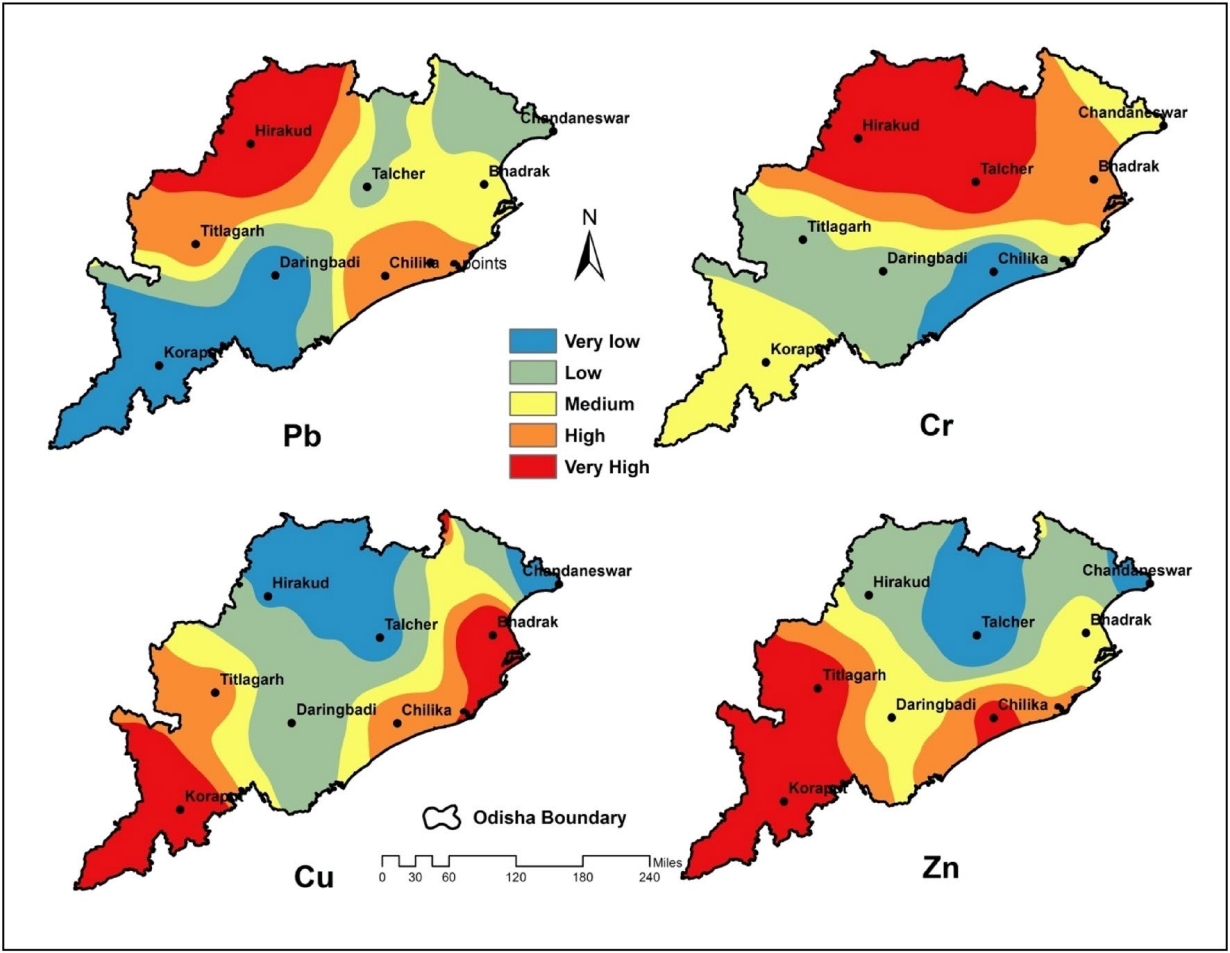
Patterns of heavy metals’ spatial distribution throughout the study area crated using ArcMap 10.2.1.

Further, when the metal contamination in the wetland soil was examined from the perspective of spatial distribution (Fig. 3), Pb decreased from the northwest to the southeast. The Cr concentration distribution was found to have a decreasing gradient from the west to the east. The distribution of Cu was recorded as increasing from the northwest to the south. The distribution pattern of Zn in soil expressed an increase towards the south from the north (Fig. 3). The threshold values of heavy metals for agricultural soils (TVAS) are given in Table 2. Comparing the detected metals with TVAS, only Cr was determined to exceed the threshold limit at all sites except Chilika^64^. All other detected metals were under the threshold limit of TVAS (Table 2). Agricultural landscapes surrounded all the sampled wetlands; therefore, comparing the heavy metal concentration with the TVAS value depicts the pollution impact. The mean Pb, Cr, and Cu values in sediment from this study area exceeded the toxicity reference value (TRV)^65^. The mean concentration of Pb overcomes the TRV at Bhadrak, Chilika, Hirakud, Talcher, and Titlagarh sites. The Cr concentration exceeded the TRV at all sampling sites. Except for Chandaneswar, all other 'sites' Cu concentrations exceeded the limit of TRV. The TRV represented the exceeding limit for heavy metal values in the region. Compared to World Common Trace Metal Range in Lakes (WCTMRL) values, the mean value of Cr was higher at Hirakud and Talcher^66^. It represented polluted conditions with high Cr concentrations among all the sampled wetlands in the study area.

Rapid urbanization, industrialization, and the developmental activity of human habitation have increased the pollutant level in the environment. The application of agrochemicals on agricultural land contributes to the increased concentration of heavy metals in bed sediments^64^. This increased pollution level ultimately moves sediments through the aquatic ecosystem^67^. This heavy metal contamination also contaminates sediment-dependent organisms. The level of heavy metals in wetlands can be assessed by detecting their concentrations in water and sediments^68^, which are found to be low in the water and high in the sediments due to accumulation^69^. The potentially harmful heavy metal in sediment is always a source of potential bioaccumulation and biomagnification^70^. Therefore, heavy metals in sediment play an indicator role in gauging environmental conditions^71^. The presence of heavy metals throughout the sediment is evidence of pollution^72^. In a given geographical framework, the distribution of spatial parameters utilizing tools like geographic information system (GIS) integrating field inventory has provided agility in scientific representation^73^. The current study expressed the distribution pattern of contaminants, and this spatial distribution represented the concentration level of heavy metals in the study area (Fig. 3).

### Element association and clustering

**Figure 4:**
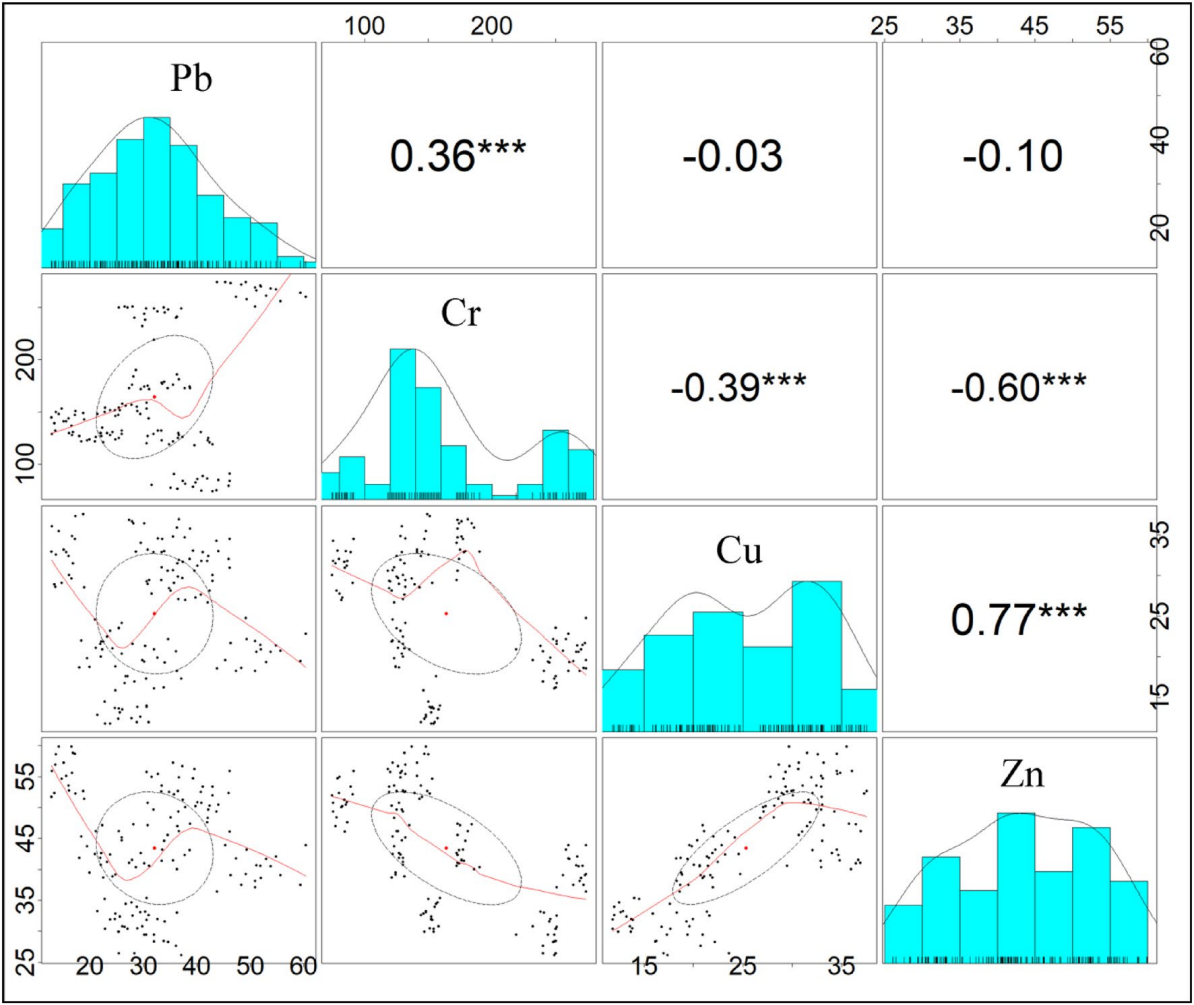
Correlogram depicting association among the heavy metals.

The association among metals was established by calculating Pearson's correlation analysis. Cu and Zn were highly positively correlated (r = 0.77). A moderately positive correlation was also found between Pb and Cr (r = 0.36). This positive correlation described a similar type of source for their emergence. The negative correlation of Cr with Cu and Zn can be associated with their related geochemical properties. This correlogram supports understanding the presence of heavy metals in the sediment (Fig. 4). Here, the strong association between Cu and Zn may be due to the binding of strong hydrated metals^51,74^. Having the same chemical characteristics, Cu and Zn show the same behavior and distribution pattern^56^. Therefore, the association of Pb and Cr may describe the higher affinity between these metals^74^.

**Figure 5:**
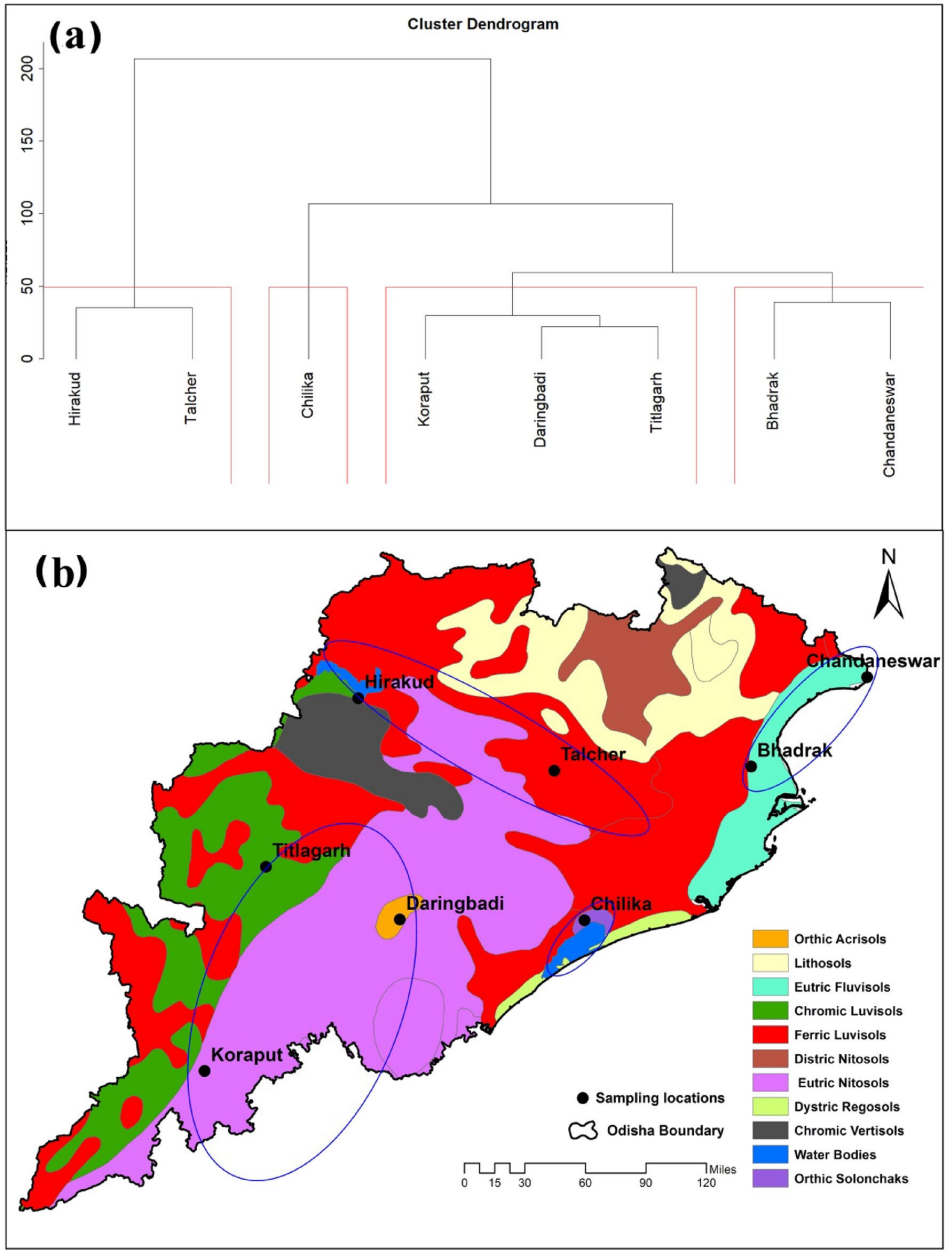
Hierarchical Cluster (**a**) of all sampling sites according to heavy metals concentration and comparison with soil types (**b**) of the study area crated using ArcMap 10.2.1^75^.

Cluster analysis was performed among the heavy metal concentrations at all sampling locations (Fig. 5). It indicated the Bhadrak and Chandaneswar sites in one Cluster. These two sampling locations are from coastal regions, and the same lithogenic soil type can be the reason for the clustering into one. This study area region has the fluvisol soil type, representing the genetically younger soil with alluvial deposits. This soil type can be found in coastal lowlands, river fans, and tidal marshes^75^. Another type of Cluster that was very similar was found at the Hirakud and Talcher sample sites. An exceptionally high Pb and Cr content was found at these two locations.

These regions of the study area were distributed with luvisols of higher clay content. This soil has a higher fertility due to its various mineral parent materials. The Koraput, Daringbadi, and Titlagarh sites comprise the southern portion of the region under investigation. The habitat and the same nitisol soil types might contribute to this clustering (Fig. 5). This soil type is mainly found in the highlands and is formed from the parent rock material. The southern region under investigation was from the Eastern Ghats mountain ranges^25,76,77^.

### Contamination indices of pollution

The contamination factor depicts the pollution and contamination levels of environmental media. Comparing the sediment concentration with the background value describes CF^55^. This background value comprises the mean international value^78^ or regional background value^79,80^. The background values of these metals here were referred to as a nationalized study on sediments^8^. The present study portrayed the Pb contamination as low at Koraput and moderated at all other sites. The Cr contamination was moderate at Chilika and considerably high at Bhadrak, Chandaneswar, Daringbadi, Koraput, and Titlagarh sites. Sediment samples from Hirakud and Talcher were highly contaminated by Cr pollution, with CF = 7.60 and 6.97, respectively. Contamination due to Cu and Zn was found to be low at all the sites, as CF < 1 (Table 4).

**Table 4:** The ecological and human health risk posed by heavy metals at all sampling sites of the study area.

Sampling sites	Pb			Cr			Cu			Zn			RI
	CF	I_geo_	PERF	CF	I_geo_	PERF	CF	I_geo_	PERF	CF	I_geo_	PERF	
Bhadrak	1.64	0.33	8.22	5.06	1.01	10.11	0.98	0.28	4.90	0.61	0.12	0.61	23.84
Chandaneswar	1.32	0.27	6.61	4.34	0.87	8.69	0.38	0.11	1.92	0.46	0.09	0.46	17.67
Chilika	2.01	0.40	10.07	2.34	0.47	4.69	0.89	0.25	4.43	0.72	0.14	0.72	19.91
Daringbadi	1.12	0.22	5.58	3.63	0.73	7.26	0.61	0.17	3.05	0.60	0.12	0.60	16.49
Hirakud	2.56	0.51	12.81	7.60	1.53	15.20	0.59	0.17	2.95	0.56	0.11	0.56	31.53
Koraput	0.78	0.16	3.89	4.03	0.81	8.06	0.93	0.26	4.65	0.78	0.16	0.78	17.38
Talcher	1.57	0.31	7.84	6.97	1.40	13.94	0.55	0.15	2.74	0.42	0.08	0.42	24.94
Titlagarh	1.83	0.37	9.16	3.57	0.72	7.14	0.86	0.24	4.28	0.74	0.15	0.74	21.32

*CF* contamination factor, *I*_geo_ Geo-accumulation index, *PERF* potential ecological risk factor, *RI* ecological risk index.

The geoaccumulation index (Igeo) calculates the study area's metal accumulation. Considering the Igeo grade depicted previously, Pb, Cu, and Zn accumulations were considered uncontaminated to moderately contaminated sediment. However, the geoaccumulation of Cr at Bhadrak, Hirakud, and Talcher was more significant than 1, so these sampling sites were moderately contaminated (Table 4).

### Ecological risk assessment

**Figure 6:**
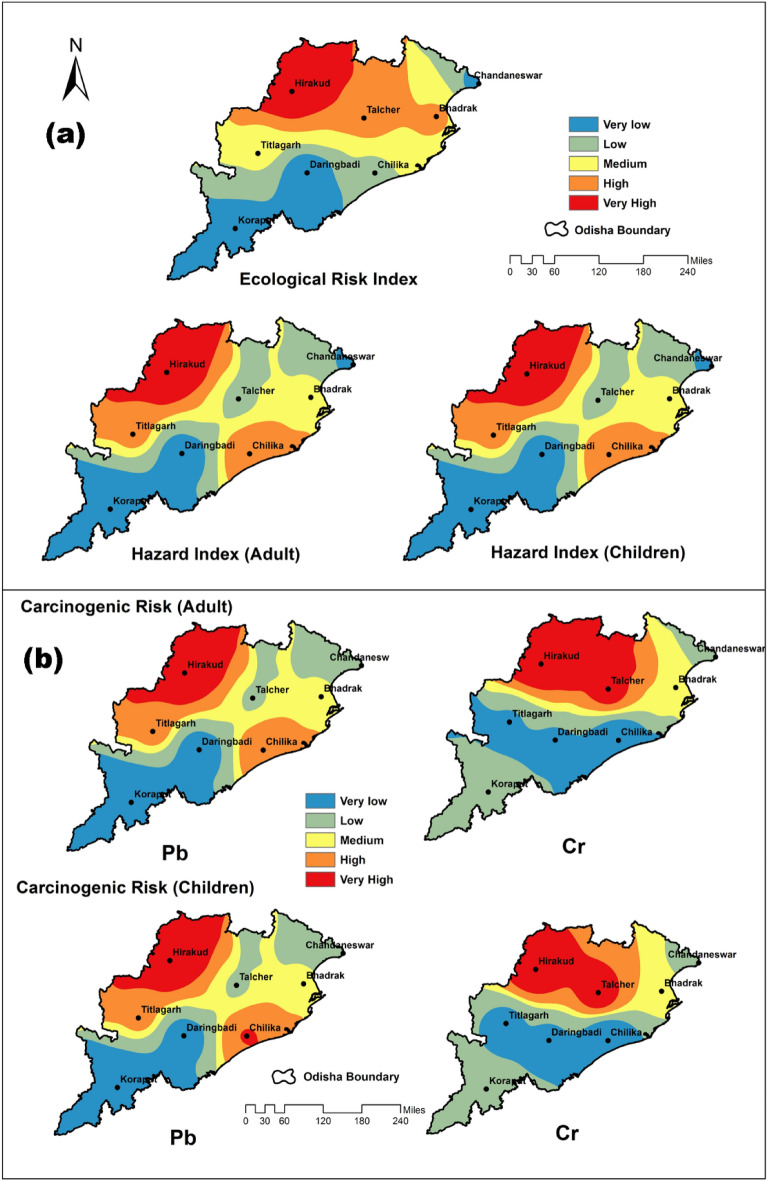
The pattern of ecological risk index, hazard index (adults and children) (**a**), and carcinogenic risk (**b**) posed by heavy metals in sediments of the study area crated using ArcMap 10.2.1.

The current research determined the potential ecological risk factor (PERF) for each type of metal across all locations. The PERF obtained by all detected heavy metals in one region can be added to achieve the ecological risk index (RI)^59^. The present study depicted a low ecological risk with the highest RI at the Hirakud sampling site (Table 4). As all sampling sites were found to have RI < 150, the region under examination may pose a negligible threat to the environment^58^. This RI is updated with all detected metals’ limits^81,82^. The gradient of ecological risk in this study area decreases towards the south from the north (Fig. 6).

### Human risk assessment

The harmful substances from sediment move into the human health system through indirect ingestion^83,84^. The present study depicted the harmful non-carcinogenic effect on humans due to indirect ingestion, as HQ values for Pb, Cr, Cu, and Zn at all the study sites were more significant than 1. This HQ value indicated a high health risk for adults and children. The only HQ of Cr at the Chilika site had a lower value than the limit for adult ingestion (Table 5). The high-end health risk of heavy metals for humans is also described by the hazard index (HI), which can be calculated from the HQ value^85^. The HI values were more significant than one, which was always considered a high health risk for adults and children^86^. The probability of chronic non-carcinogenic effects grows in proportion to the number in HI value^58^. The HI value in the sediments of the entire sampling site in this investigation showed that it was much greater than the threshold level (HI < 1) (Table 5). It indicates increased danger to human health in the region being studied. The pattern of HI can be seen lower in the southern part of the study area. In contrast, the north-western part depicts the high HI in adults and children (Fig. 6). Oral exposure by ingesting food contaminated with heavy metals from the sediments of this area can have long-term impacts that are not cancer-causing.

**Table 5:** Average daily dose (ADD), hazard quotient (HQ), and hazard index (HI) of different heavy metals at different sampling sites of the study area.

Sampling sites	Pb				Cr				Cu				Zn				HI_A_	HI_C_
	ADD_A_	ADD_C_	HQ_A_	HQ_C_	ADD_A_	ADD_C_	HQ_A_	HQ_C_	ADD_A_	ADD_C_	HQ_A_	HQ_C_	ADD_A_	ADD_C_	HQ_A_	HQ_C_		
Bhadrak	0.49	0.66	140.91	187.88	2.66	3.54	1.77	2.36	0.51	0.69	12.85	17.13	0.65	0.86	2.16	2.88	157.69	210.26
Chandaneswar	0.40	0.53	113.33	151.10	2.28	3.04	1.52	2.03	0.20	0.27	5.04	6.72	0.49	0.65	1.63	2.17	121.51	162.01
Chilika	0.60	0.81	172.70	230.26	1.23	1.64	0.82	1.09	0.47	0.62	11.63	15.50	0.76	1.02	2.55	3.40	187.69	250.26
Daringbadi	0.33	0.45	95.65	127.54	1.91	2.54	1.27	1.69	0.32	0.43	8.02	10.69	0.64	0.85	2.13	2.85	107.08	142.77
Hirakud	0.77	1.03	219.65	292.87	3.99	5.32	2.66	3.55	0.31	0.41	7.74	10.32	0.60	0.80	2.00	2.67	232.05	309.40
Koraput	0.23	0.31	66.64	88.86	2.12	2.82	1.41	1.88	0.49	0.65	12.21	16.28	0.83	1.11	2.77	3.70	83.03	110.71
Talcher	0.47	0.63	134.34	179.12	3.66	4.88	2.44	3.25	0.29	0.38	7.19	9.59	0.45	0.59	1.49	1.98	145.46	193.94
Titlagarh	0.55	0.73	157.00	209.34	1.88	2.50	1.25	1.67	0.45	0.60	11.22	14.96	0.79	1.06	2.64	3.52	172.12	229.49

*ADD*_A_ average daily dose for an adult, *ADD*_C_ average daily dose for children, *HQ*_A_ hazard quotient for adults, *HQ*_C_ hazard quotient for children, *HI*_A_ hazard index for adults, *HI*_C_ hazard index for children.

The carcinogenic risk (CR) value < 1 × 10^–6^ can be considered having no effect, and between 1 × 10^–6^ and 1 × 10^–4^ represents the endurable limit for human beings^87^. This carcinogenic risk calculated in the current investigation was only for the ingestion pathway, which means the accumulation of elements/heavy metals in food from the sediment ultimately leads to cancer in human beings^8^. All locations where samples were collected from the study area had a carcinogenic risk higher than the threshold limit for Cr. Cu and Zn were not listed due to their non-carcinogenic effects. However, a higher concentration of these two elements can cause endocrine disruption and various chronic diseases in humans^88^. Previously, one chromite mining location in the study area had explained the carcinogenic effect due to the ingestion of plant parts^35^. Of all the locations, Hirakud possesses the highest CR in adults and children (Table 6).

**Table 6:** Carcinogenic risks from different sites of the study area.

Sampling sites	Pb		Cr	
	CR_A_	CR_C_	CR_A_	CR_C_
Bhadrak	0.0042	0.0056	1.3275	1.7700
Chandaneswar	0.0034	0.0045	1.1400	1.5200
Chilika	0.0051	0.0069	0.6153	0.8205
Daringbadi	0.0028	0.0038	0.9525	1.2700
Hirakud	0.0065	0.0087	1.9950	2.6600
Koraput	0.0020	0.0026	1.0575	1.4100
Talcher	0.0040	0.0053	1.8300	2.4400
Titlagarh	0.0047	0.0062	0.9375	1.2500

*CR*_A_ carcinogenic risks by ingestion in adults, *CR*_C_ carcinogenic risks by ingestion in children.

Since the water from these wetlands is not being drawn directly for human consumption, the only way for people in the surrounding community to indirectly consume it is by consuming various foods from that wetland, such as fish, rice, some vegetables, and spinach. The CR in adults and children caused by indirect ingestion of Pb can be seen decreasing towards the south from the western region. The carcinogenic risk due to Cr ingestion can be depicted as higher in the northern half and lower in the southern portion of the region under investigation (Fig. 6). The districts of western Odisha had been recorded as having the highest number of cancer patients among all the districts^89^, supporting current research. Bargarh, Sambalpur, and Bolangir districts of the western side of the investigation region have the highest percentage of recorded patients among all the districts (26.34, 24.58, and 10.81, respectively)^89^. The exposure time to these heavy metals can be a significant factor, as the highest numbers of patients are detected in the 40–60 age group^89^.

The higher concentration of heavy metals in soils is transferred to edible plants and pesticides that humans ingest and ultimately possess carcinogenic effects^90,91^. Industrial development in the study area also poses carcinogenic effects due to the addition of heavy metals in soil from the effluents^92^. The western part of the study area is a hub for rice production^93^. The contamination of rice grains due to contaminated soils has been documented in previous investigations^94,95^, and the use of pesticides also increases the carcinogenic risk sometimes^91^. The local community faces a significant danger to their health if they consume any of this infected rice^96^ as it has already been recorded in different rice species in previous studies from this region^47,49,50^. This could be one of the reasons for the increasingly higher number of cancer patients in the particular region of the study area, which is supported by previous studies^91,97^. Considering the present scenario, this research paper offers some background information on the accumulation of heavy metals in wetland sediments and their carcinogenic effects on human beings. The significance of the current study lies in the fact that it protects the human population and the environmental ecosystem by assessing the potential risks to human health. This study's significance to the region's population stems from the fact that the carcinogenic and non-carcinogenic dangers posed by heavy metal contamination in the environment are considered. Because pollution from heavy metals is a problem affecting the entire developing world, this situation may also represent a worldwide picture. This information could serve as a foundation for formulating successful policies, raising awareness, and creating a future that is both healthy and sustainable.

## Conclusions

The levels of metals like Pb, Cr, and Cu found in the investigation region exceeded the toxicity reference value in sediments. The concentration of heavy metals above the threshold limit can be directly linked to the food chain through plant uptake. The natural wetlands had lower Cr, Zn, and Pb, while the constructed wetlands had higher Cr, followed by Pb and Zn. The high contamination of heavy metals poses an ecological risk to the wetlands, leading to human health risks in these regions. The hazard index higher than the threshold for adults and children is the health risk from polluted sediments. Pb and Cr contamination pose a carcinogenic effect on humans and can cause cancer in the study area. Heavy metal contamination in sediments in India's wetlands can have significant environmental and health hazards. The contamination can negatively impact the biodiversity of the wetland ecosystem and potentially harm animals and plants that live in and around the wetland. Heavy metals in sediment can also pose a cancer-causing risk to human health for those who come into contact with the contaminated sediments or consume fish and other aquatic life from the wetland. It is essential for proper monitoring and management of these wetlands to take place to mitigate these hazards.

## Data Availability

The datasets generated and analysed during the study are available from Bibhu Prasad Panda (lead author, bibhuprasadpanda14@gmail.com) and Hemen Sarma (corresponding author, hemens02@yahoo.co.in) on reasonable request.
